# Iatrogenic left ventricular-right atrial communication after tricuspid annuloplasty; a case report

**DOI:** 10.1186/1749-8090-9-104

**Published:** 2014-06-18

**Authors:** Eiki Tayama, Yukihiro Tomita, Ken-ichi Imasaka, Takanori Kono

**Affiliations:** 1 Department of Cardiovascular Surgery, Clinical Research Institute, National Hospital Organization Kyushu Medical Center, 1-8-1 Jigyohama, Chuo-ku, Fukuoka 810-8563, Japan

**Keywords:** Tricuspid annuloplasty, Complication, Left ventricular-right atrial communication

## Abstract

A 75-year-old man (Asian, Japanese) was readmitted for examination of a heart murmur and haemolytic anemia 3 months after mitral valve and tricuspid annuloplasties and coronary artery bypass. A new systolic murmur was heard, and echocardiography showed a high-velocity jet originating from the left ventricular outflow tract and extending to the right atrium, a small defect between the left ventricle and the right atrium. No periprosthetic leaks were found in the mitral position. We judged that surgical repair of the defect was essential to treat mechanical haemolysis. At operation, we found a communication (3 mm in diameter) just beneath the detached prosthetic ring at the anteroseptal commissure of the tricuspid valve. After partially removing the tricuspid ring from the anteroseptal commissure area, the defect was closed using a single mattress suture with pledget. In this case, the tricuspid annuloplasty stitch in the atrioventricular region was probably placed on the membranous septum rather than on the tricuspid annulus. A tear then occurred in the atrioventricular membranous septum, leading to left ventricular–right atrial communication.

## Background

Acquired left ventricular-right atrial (LV-RA) communication following aortic or mitral valve replacement is reported to occur infrequently [1-6], but LV-RA communication can also result from tricuspid annuloplasty (TAP) [7,8]. We present a case of iatrogenic LV-RA communication associated with TAP with a rigid prosthetic ring.

## Case presentation

**Figure 1:**
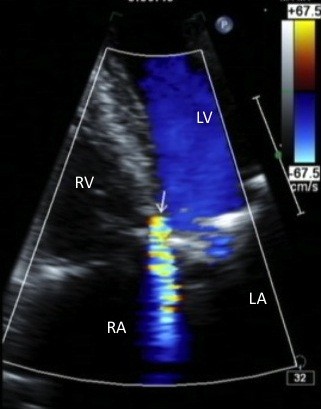
**Preoperative echocardiography showing a high-velocity jet (arrow) originating from the left ventricular outflow tract and extending to the right atrium along the tricuspid valve leaflet.** LA: left atrium; LV: left ventricle; RA: right atrium; RV: right ventricle; TV, tricuspid valve.

**Figure 2:**
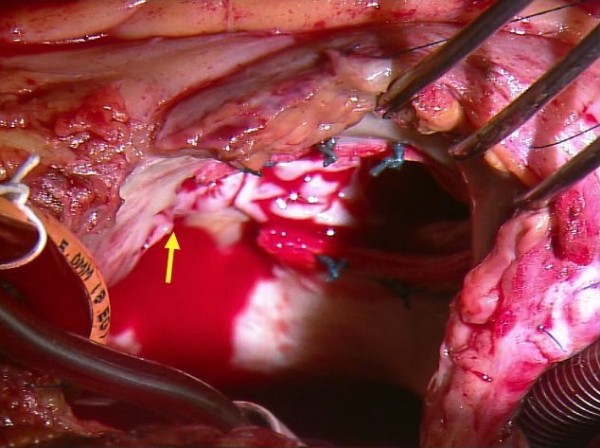
**Operative findings.** After partially removing the detached rigid tricuspid annuloplasty ring, a 3 mm defect was found in the atrial septum close to anteroseptal commissure of the tricuspid annulus. Bright red blood entering the right atrium through this defect from the left ventricle.

A 75-year-old man (Asian, Japanese) was admitted to our hospital for examination of a heart murmur and haemolytic anemia. The patient had a history of mitral valve annuloplasty with a Carpentier-Edwards Physio II Annuloplasty Ring, 30-mm (Edwards Lifesciences Corp., Irvine, CA, USA), TAP with a rigid prosthetic ring (Edwards MC^3^ Tricuspid Annuloplasty Ring, 32 mm; Edwards Lifesciences Corp, Irvine, CA, USA), and coronary artery bypass 3 months previously. At the initial operation, no debridement of the mitral annulus had been performed in the posteromedial commissure or vicinity. Postoperative echocardiography performed 1 month after the operation showed no mitral regurgitation and trace tricuspid regurgitation.On admission, a high-pitched pansystolic murmur was audible along the left sternal border. Laboratory studies showed decreased haemoglobin (6.7 g/dL), elevated serum lactate dehydrogenase (2,211I U/L), and fragmentation of red blood cells. Echocardiography showed a high-velocity jet originating from the LV outflow tract and extending to the RA along the tricuspid valve leaflet through a small defect (Figure 1). However, no leaks were detected in the mitral position. Cardiac catheterization and left ventriculography confirmed LV-RA communication. We judged that surgical repair of the LV-RA communication was essential to treat mechanical haemolysis, regardless shunt amount.At operation, the patient was noted to have a communication just beneath the detached prosthetic ring at the anteroseptal commissure of the tricuspid valve, and a jet of bright red blood entering the right atrium through the defect (Figure 2). Two previous TAP mattress sutures were removed, and the detached rigid TAP ring was then cut and removed 1 cm from left-side edge of the ring. A 3-mm defect was found in the atrial septum adjacent to the tricuspid annulus. The defect was repaired with a pledgeted mattress suture of 2-0 polyester (Nespolene, Alfresa Pharma Co., Tokyo, Japan) by passing the needle from the right ventricle to the right atrium posterior to the tricuspid valve. In addition, the uncovered cut edge of the TAP ring was covered with a pledgeted mattress suture of 4-0 polypropylene (Prolene™, Ethicon Endo-Surgery, Inc., Blue Ash, OH, USA). The remaining section of the ring was left in place. Intraoperative transesophageal echocardiography showed no residual LV-RA shunt. The patient has been asymptomatic for more than 10 months after the operation, with trivial tricuspid valve regurgitation and no signs of residual left-to-right shunt or haemolysis.

### Discussion

LV-RA communication has been noted to occur rarely after aortic or mitral valve replacement [1-6]. While these procedures, extended debridement of annular calcifications in the posteromedial commissure and its vicinity may lead to membranous septum injury and LV-RA communication [1,2]. However, no annular debridement in these areas had been performed in our patient. Furthermore, the location of the communication in this case differed from that of LV-RA communication following mitral surgery, in which case the location of the defect is usually at the base of the septal tricuspid leaflet near the coronary sinus.

Iatrogenic LV-RA communication caused by TAP suturing has been also reported, but it is extremely rare [7,8]. Anatomically, the line of attachment of the septal leaflet of the tricuspid valve crosses the membranous septum, dividing it into an anterior interventricular portion, which lies between the ventricles, and a posterior atrioventricular portion, which lies between the left ventricle and right atrium. In addition, the position of the mitral annulus is slightly more cephalad than that of the tricuspid valve. Trauma to the membranous septum could therefore result in either a ventricular septal defect or an LV-RA communication. According to Aoyagi et al., if a mattress suture for TAP is inserted into the atrioventricular portion of the membranous septum rather than the tricuspid annulus at the anteroseptal commissure of the tricuspid valve, dehiscence of the ring and creation of a tear of the atrioventricular membranous septum may occur [7]. We speculated that a similar phenomenon had occurred in our case. Interestingly, all previously reported LV-RA communication after TAP, including the present case, used a rigid ring [7,8], suggesting that strong tension around the tricuspid annulus associated with a rigid ring may lead to increased risk for LV-RA communication.

## Conclusion

We report a rare case of iatrogenic LV-RA communication occurring after TAP with a rigid prosthetic ring. We emphasize that LV-RA communication is associated not only with aortic or mitral valve replacement, but also with TAP.

## Consent

Written informed consent was obtained from the patient for publication of this Case Report and any accompanying images. A copy of the written consent is available for review by the Editorial-in-Chief of this journal.

## Abbreviations

LV-RA: Left ventricular-right atrial; TAP: Tricuspid annuloplasty.

## Competing interests

The authors declare that they have no competing interests.

## Authors’ contributions

ET wrote the draft of the manuscript. YT performed literature review. KI and TK obtained the data and written consent. All authors have read and approved the final manuscript.
